# Gastrointestinal Basidiobolomycosis in Pediatric Cases With Multiple-Site Involvement: A Case Series

**DOI:** 10.7759/cureus.76451

**Published:** 2024-12-27

**Authors:** Mohaned Mohammed, Ahmed Albishri, Ali Alabbas, Sami E Abdelmogeit, Badriah G Alasmari, Jameelah A Alqahtani, Samah E Mohammed, Ali Hawan, Mahmoud Hussein, Muhammad Saeed, Yassin Hamid, Eman Ghazwani

**Affiliations:** 1 Pediatrics, Armed Forces Hospital Southern Region, Khamis Mushait, SAU; 2 Pediatric Infectious Diseases, Armed Forces Hospital Southern Region, Khamis Mushait, SAU; 3 Pediatrics, Najran General Hospital, Najran, SAU; 4 Pediatric Hematology &amp; Oncology, Armed Forces Hospital Southern Region, Khamis Mushait, SAU; 5 Pathology, Armed Forces Hospital Southern Region, Khamis Mushait, SAU; 6 Pediatric Neurology, Armed Forces Hospital Southern Region, Khamis Mushait, SAU; 7 Pediatric Gastroenterology, Armed Forces Hospital Southern Region, Khamis Mushait, SAU

**Keywords:** basidiobolomycosis, basidiobolus ranarum, git basidiobolomycosis, itraconazole, southern region saudi arabia

## Abstract

Basidiobolomycosis, a rare fungal infection seen in immunocompetent patients, is a chronic granulomatous infection affecting the skin and subcutaneous tissue. It is caused by the fungus *Basidiobolus ranarum*. Gastrointestinal basidiobolomycosis usually has non-specific clinical manifestations, and its diagnosis requires a high index of suspicion. In pediatric patients, any delay or misdiagnosis may lead to morbidity and or mortality. Herein, we report three cases of gastrointestinal basidiobolomycosis from the southern region of Saudi Arabia. The three patients were diagnosed based on histopathology results and computed tomography of the abdomen, with different pathological sites with multiple gastrointestinal tract involvement. Case one was a five-year-old male patient complaining of abdominal pain lasting seven months, weight loss, and fever, with computed tomography (CT) showing involvement of the liver and bowels. Case two was a five-year-old male patient complaining of a fever lasting one month associated with generalized abdominal pain, with CT abdomen showing involvement of the liver, colon, cecum, and mesenteric lymph node. Case three was a seven-year-old female patient complaining of abdominal pain lasting one month, with a CT abdomen showing involvement of the colon and terminal ilium accompanied by mesenteric lymph node involvement. In all three cases, the CT abdomen findings showed involvement of the bowel. All three cases were treated successfully with antifungal monotherapy (itraconazole) without surgical intervention, and all responded well. The aim of this case series was to show the importance of early management for improved outcomes in such cases and to highlight how a diagnosis of gastrointestinal basidiobolomycosis requires a high index of suspicion due to its broad range of clinical presentation. In addition, medical management with itraconazole can be curative without the need for surgical intervention.

## Introduction

Basidiobolomycosis, an enigmatic and uncommon fungal infection clinically and radiologically, is caused by *Basidiobolus ranarum (B. ranarum)* and is reported in immunocompetent individuals [1]. Globally, Saudi Arabia has the highest overall incidence of gastrointestinal basidiobolomycosis [2]. In 1955, *B. ranarum* was first isolated from decaying plant material in the United States [3]. In Indonesia, the first recognized human case of *B. ranarum* infection was reported in 1956, presenting as subcutaneous mycosis [4]. The first culture-positive case of invasive basidiobolomycosis of the maxillary sinus and palate was reported in a 4-year-old boy from the United States in 1978 [5].

*B. ranarum* is an environmental saprophyte and a member of the order Entomophthorales of the class Zygomycete [3]. *B. ranarum* is found in decaying vegetable materials and surrounding soil, as well as the gastrointestinal tract (GIT) of amphibians, reptiles, dogs, frogs, and bats. It usually infects subcutaneous tissues and can affect visceral organs causing significant disease, but it very rarely involves systems apart from the GIT [6]. As a rare disease, diagnosis of gastrointestinal basidiobolomycosis requires a high index of suspicion, with risk factors and clinical presentation that resemble other diseases, such as lymphoma, inflammatory bowel disease, and other GIT infections.

Herein, we report three cases of gastrointestinal basidiobolomycosis by presenting their clinical features, histopathological findings, and radiological findings. These three cases showed multiple-site involvement and were treated successfully with a single antifungal agent (itraconazole) without surgical intervention. We also discuss the relevant literature for these cases.

## Case presentation

Case 1

A five-year-old male patient from Jizan (southern region of Saudi Arabia) presented to our hospital with complaints of intermittent generalized abdominal pain lasting seven months associated with weight loss, fever with night sweating for one month, anorexia, and decreased appetite. On examination, his abdomen was soft and lax, with right upper quadrant tenderness, and no organomegaly; systemic examination other than that mentioned above was unremarkable. Laboratory tests revealed microcytic hypochromic anemia, leukocytosis, eosinophilia, and thrombocytosis with elevated C-reactive protein (CRP) and erythrocyte sedimentation rate (ESR) (Table 1).

**Table 1:** Lab results showing microcytic hypochromic anemia, leukocytosis, eosinophilia, and thrombocytosis with elevated ESR and CRP. TWBC: total white blood cells, CRP: C-reactive protein, ESR: erythrocyte sedimentation rate.

Lab test	Lab result	Reference range
Hemoglobin	9.5 g/dL	10-14 g/dl
TWBC	21.4 × 10^9 ^/L	6-17 × 10^9^ /L
Eosinophil %	21.6%	-
Absolute eosinophilia	4.54 × 10^9 ^/L	0.2-1.9 × 10^9 ^/L
Platelets	678 × 10^9^/L	150 - 450 × 10^9^/L
CRP	192.7 mg/L	<5 mg/L
ESR	120 mm/h	<10 mm/h

**Figure 1:**
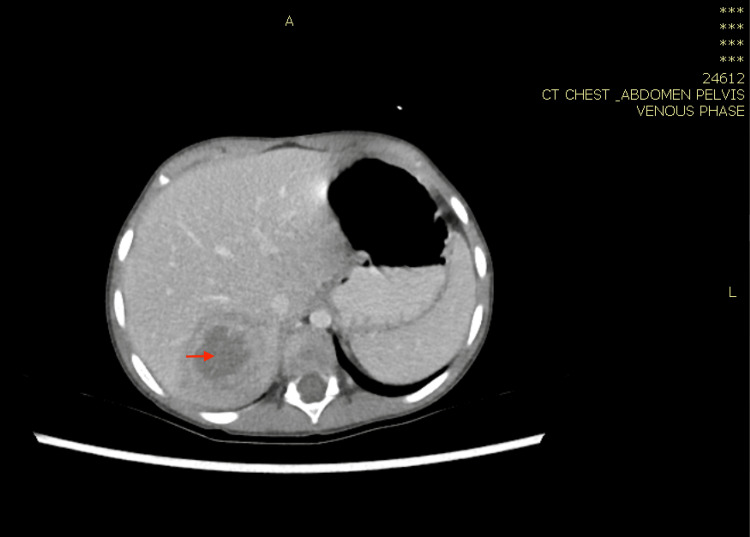
Computed tomography of the chest showing fairly well defined peripherally enhanced, centrally hypoattenuating lesion around 45 Hu in the 6th to 7th liver segments with a thick, irregular wall measuring 3.8 cm × 4 cm × 2.8 cm associated with surrounding peripheral edema (red arrow).

**Figure 2:**
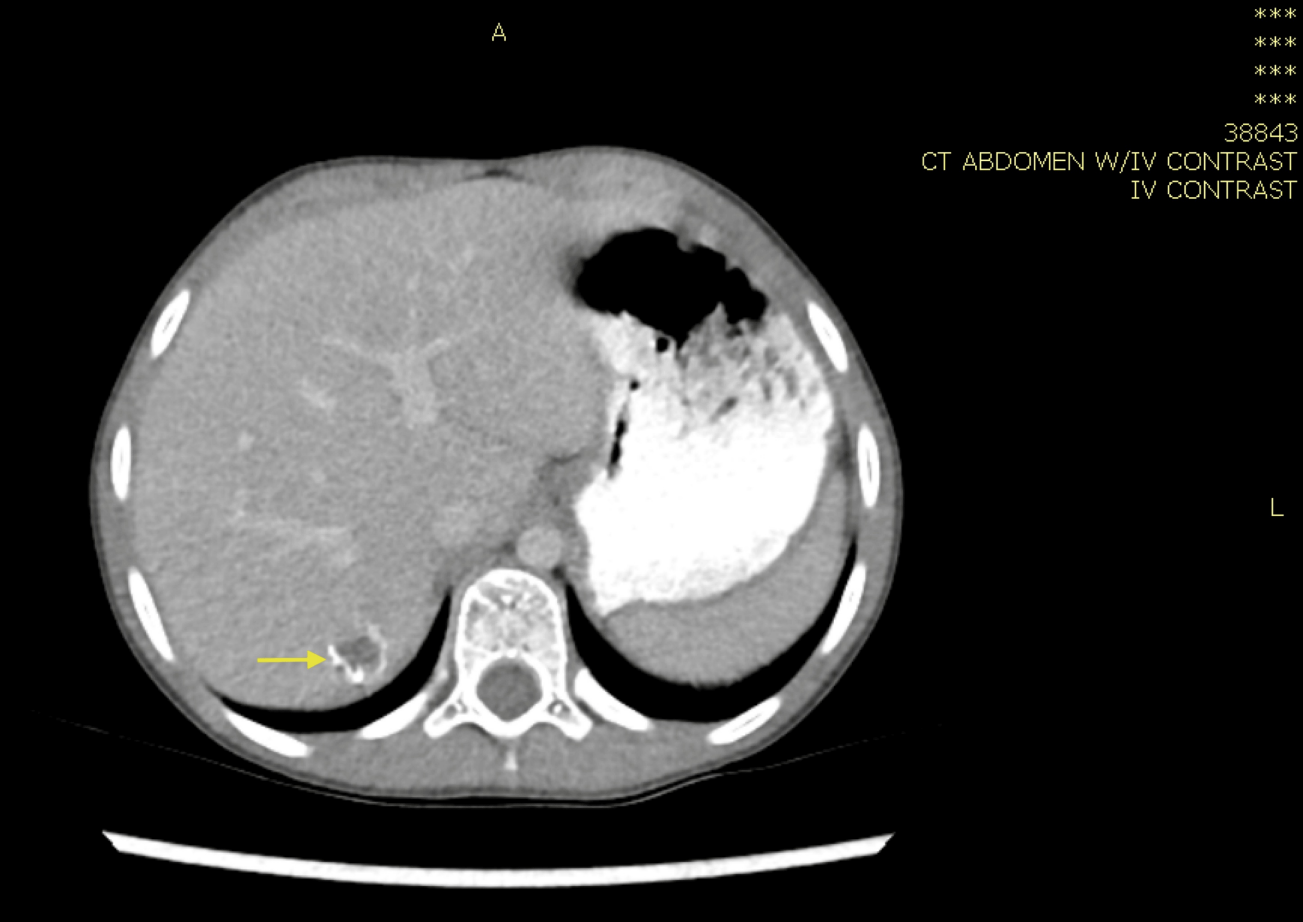
Computed tomography of the abdomen showing liver with normal size and homogenous enhancement with no obvious underlying parenchymal disease. Significant circumferential wall thickening was observed involving the ascending colon (yellow arrow), hepatic flexure, and mesentery lymphadenopathy, with impression of basidiobolomycosis fungal infection with right-sided bowel wall thickening and hepatic abscess formation.

The patient was admitted for further investigation to exclude malignancy or abdominal tuberculosis. Ultrasound abdomen showed a hyperechoic lesion with a hypoechoic rim in the right lobe of the liver (span: 101 mm) measuring 47x33 mm. CT scan of the abdomen with IV contrast showed a fairly well-defined lesion in the liver and significant circumferential wall thickening was observed involving the ascending colon (Figures 1, 2).

**Figure 3:**
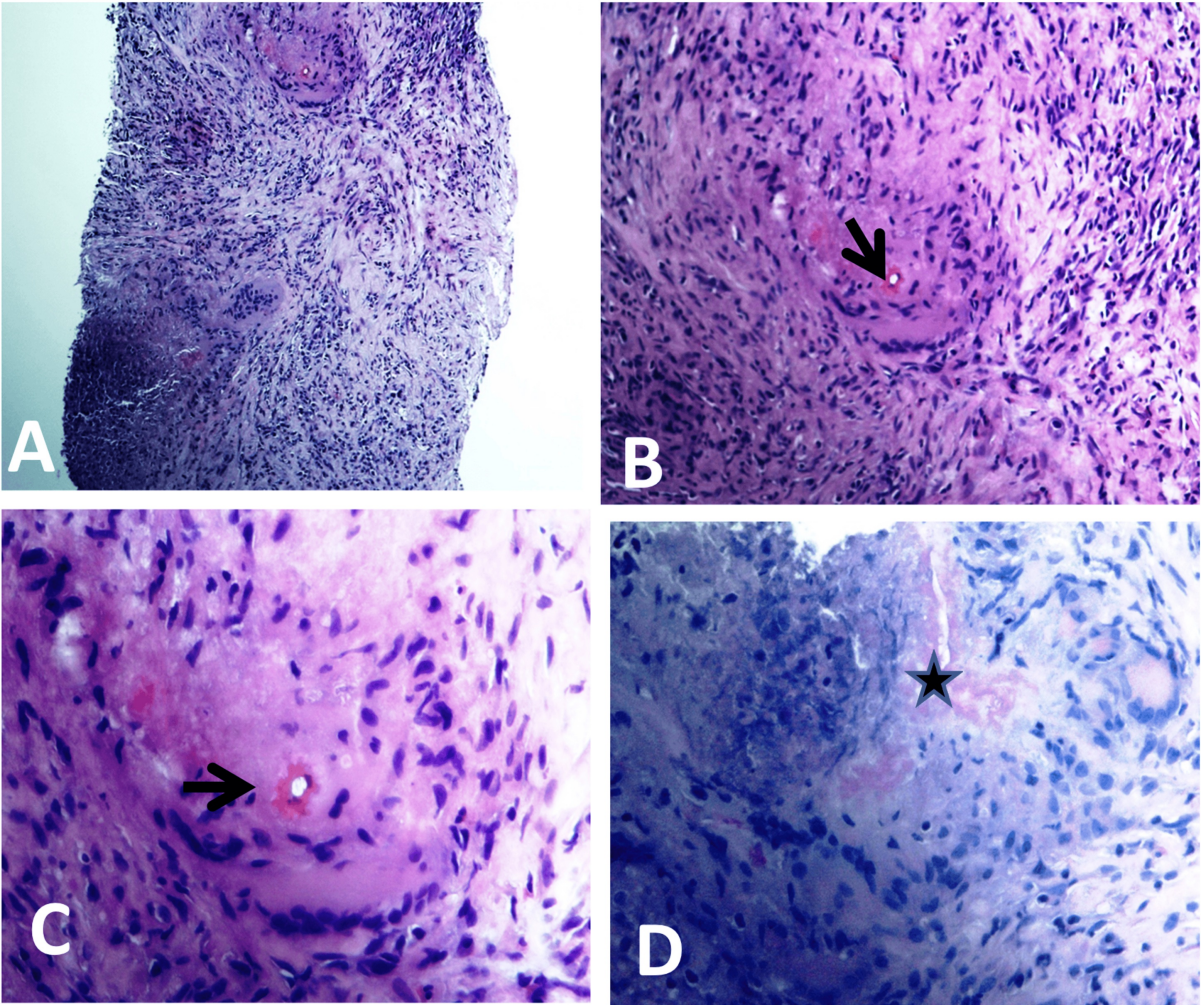
(A–D) Basidioblomycosis of the liver. Routinely stained histological sections show liver and fibroconnective tissues with non-necrotizing eosinophil-rich granulomatous inflammation. The constituent cells include mixed granulocytes (lymphocytes, histiocytes, and giant cells) and epithelioid cells with giant cells (granulomatous inflammation). Few fungal elements with thin walls (periodic acid Schiff stains; arrows in A–C) intensified the appearance of the fungal walls. Splendore–Hoeppli phenomenon (black star in D) is evident by the presence of brightly eosinophilic materials surrounding the hyphae in a starburst pattern. Magnification: (A) x200, (B) x400, (C) x200, (D) x400.

Computed tomography (CT)-guided biopsy of the colonic mass revealed fungal elements upon periodic acid Schiff (PAS) staining (Figure 3), diagnostic of gastrointestinal basidiobolomycosis of the liver and bowel wall.

**Figure 4:**
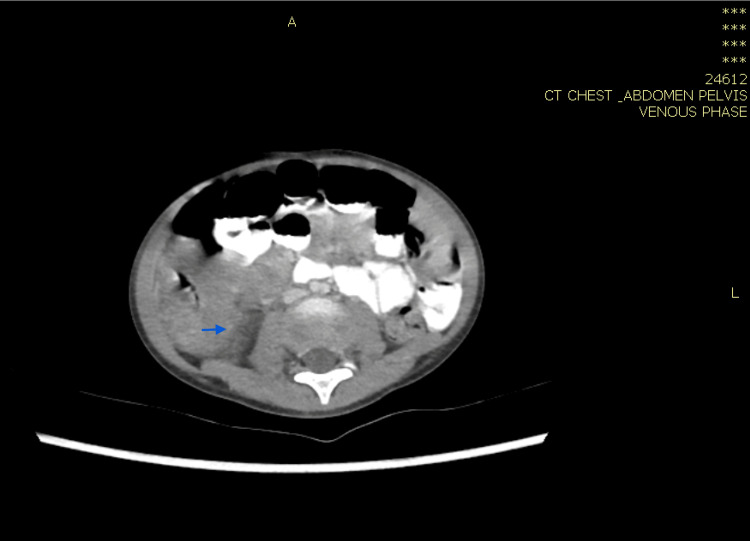
Computed tomography scan of the abdomen with IV contrast showing average liver size and well-defined marginally calcified lesion approximately 1.8 cm in the posterior segment of the right lobe, consistent with calcified granuloma. Healing of the previously noted hepatic abscesses (blue arrow) was observed.

**Figure 5:**
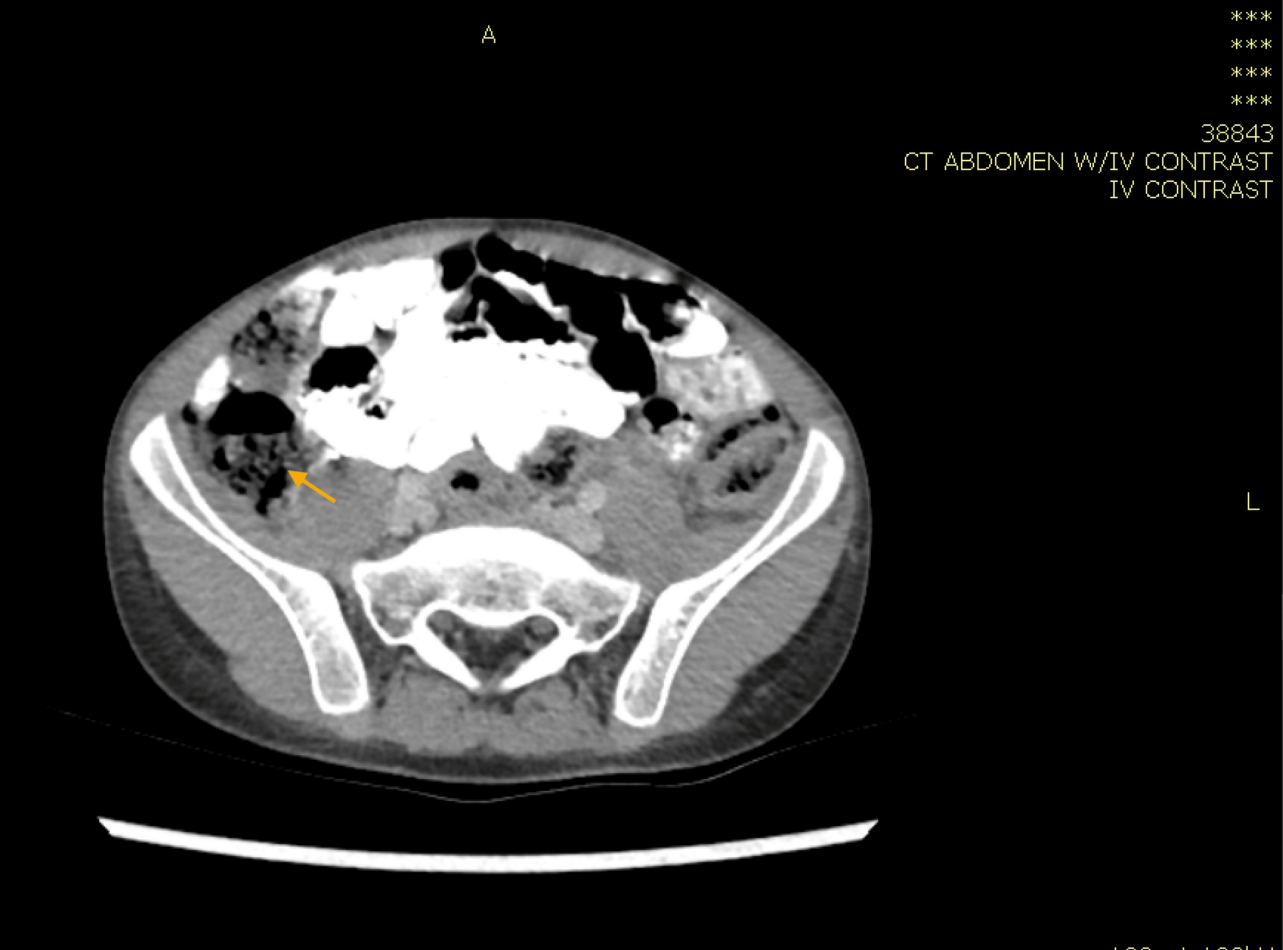
Computed tomography scan of the abdomen with IV contrast revealing no evident bowel wall thickening (orange arrow) and no further hepatic lesions. The patient was put on regular radiological and clinical follow up every three months with no features of relapse.

The patient was started on IV voriconazole for two weeks and showed significant clinical improvement. His fever and abdominal pain subsided with the normalization of laboratory parameters. He was then shifted to a full therapeutic dose of oral itraconazole for one year with regular follow-up in our clinic, as well as follow-up CT scan of the abdomen with IV contrast (Figures 4, 5).

Case 2

A five-year-old male patient from Jizan (southern region of Saudi Arabia) who was previously healthy was brought to our department by his parents with complaints of fever for one month spiking on a daily basis, generalized abdominal pain, vomiting, decreased appetite, and weight loss of 4 kg in one month. His medical and surgical histories were unremarkable. On examination, his weight was 13.8 kg (third centile). Abdominal examination revealed a distended abdomen, right hypochondrial tenderness, hepatomegaly 4 cm below the costal margin, and no splenomegaly or lymphadenopathy with unremarkable other systemic examination. Hematological investigations showed microcytic hypochromic anemia, leukocytosis, eosinophilia, and thrombocytosis with elevated ESR and CRP (Table 2).

**Table 2:** Lab results showing microcytic hypochromic anemia, leukocytosis, eosinophilia, and thrombocytosis with elevated ESR and CRP. TWBC: total white blood cells, CRP: C-reactive protein, ESR: erythrocyte sedimentation rate.

Lab test	Lab result	Reference range
Hemoglobin	9.5 g/dL	10-14 g/dl
TWBC	31.3 × 10^9 ^/L	6-17 × 10^9^/L
Eosinophil %	12.3%	-
Absolute eosinophilia	3.85 × 10^9 ^/L	0.2 - 1.9 × 10^9^/L
Platelets	819 × 10^9^/L	150 - 450 × 10^9^/L
CRP	335.4 mg/L	<5 mg/L
ESR	115 mm/h	<10 mm/h

**Figure 6:**
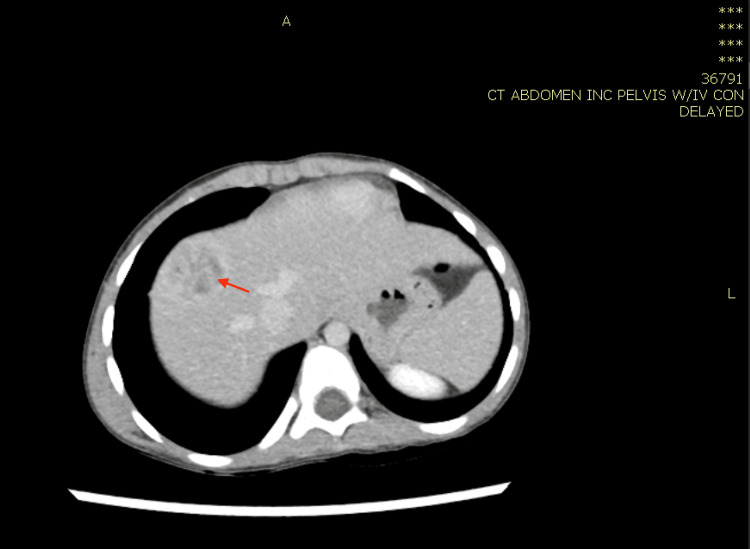
Computed tomography scan of the abdomen with IV contrast showing enlarged liver with large, ill-defined mass lesion of the medial segment of the left hepatic lobe measuring approximately 6 cm, mildly hypodense to isodose (red arrow).

**Figure 7:**
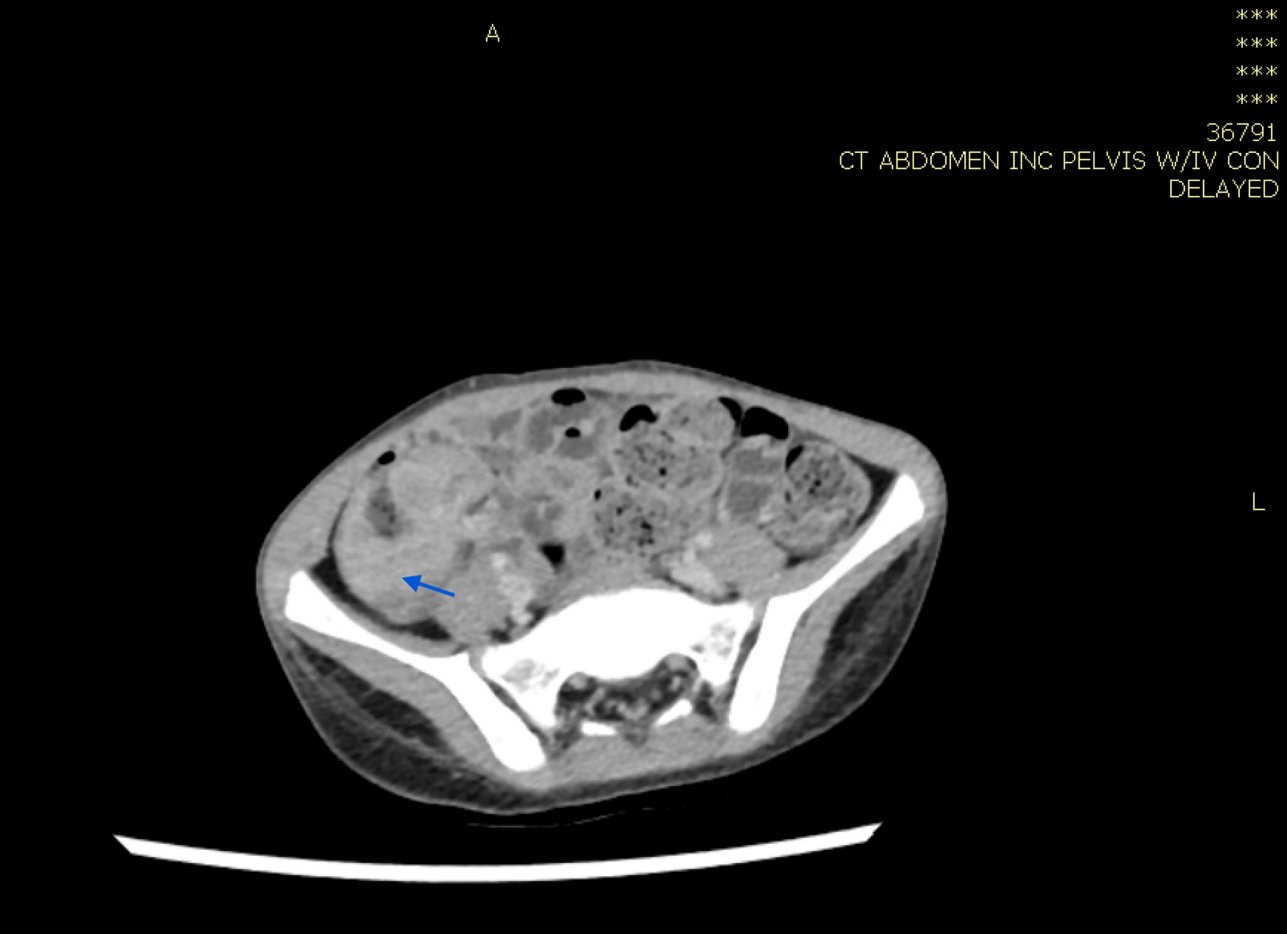
Computed tomography scan of the abdomen with IV contrast showing caecum and proximal ascending colon presenting segments of circumferential moderate wall thickening of approximately 5 cm with significant submucosal oedema and lumen narrowing (blue arrow). Multiple significantly enlarged mesenteric lymph nodes are observed, the largest of which being 1.5 cm in short diameter and 2.3 cm in long diameter, as well as two large focal lesions of the right and left hepatic lobes, associated circumferential thickening and edema of the caecum and proximal ascending colon, and pathologically enlarged necrotic lymph node at the porta hepatis and bowel mesentery. The described findings are suggestive of infection with a high index of suspicion of fungal infection, especially gastrointestinal basidiobolomycosis.

The patient was admitted to the pediatric ward as a case of pyrexia of unknown origin for further workup, including a CT scan of the abdomen with IV contrast (Figures 6, 7).

**Figure 8:**
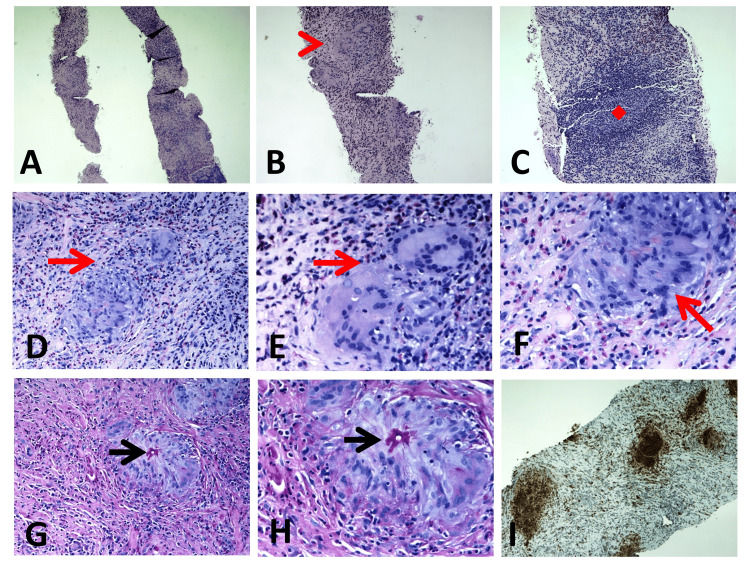
Basidioblomycosis of the retroperitoneal tissues with occurrence of basidioblomycosis within the retroperitoneal tissues: (A-C) histological sections stained with Hematoxylin and Eosin revealing areas of significantly inflamed fibroconnective tissue characterized by epithelioid cell granulomatous inflammation (red arrowhead in panel B). The infiltrate, rich in eosinophils, comprised lymphohistiocytic cells, epithelioid cells, and lymphoid follicles featuring germinal centers (red quad arrow in panel C); (D-F) presence of multinucleated giant cells (red arrows) alongside a dense eosinophilic infiltrate; (G and H) periodic acid-Schiff staining with enhanced visibility of fungal hyphae walls and surrounding eosinophilic, homogeneous, structureless material, which is indicative of the Splendore–Hoeppli phenomenon (black arrows); (I) immunohistochemical analysis results revealing dense patchy aggregates of histiocytes, identified through CD68 immunostaining. Magnification: A: x40, B-C: x100, D-G: x200, and I: x400.

Ultrasound (US)-guided true cut biopsy was taken from the solid lesion of the right hepatic lobe, with the section showing liver parenchyma with eosinophilic-rich mixed inflammation, necrosis, and few fungal elements (hyphae) with variable thickness (periodic acid-Schiff with diastase or PASD staining) surrounded by histiocytic reaction with giant cell formation. PASD staining was positive for fungal elements. These results were diagnostic for gastrointestinal basidiobolomycosis (Figure 8).

**Figure 9:**
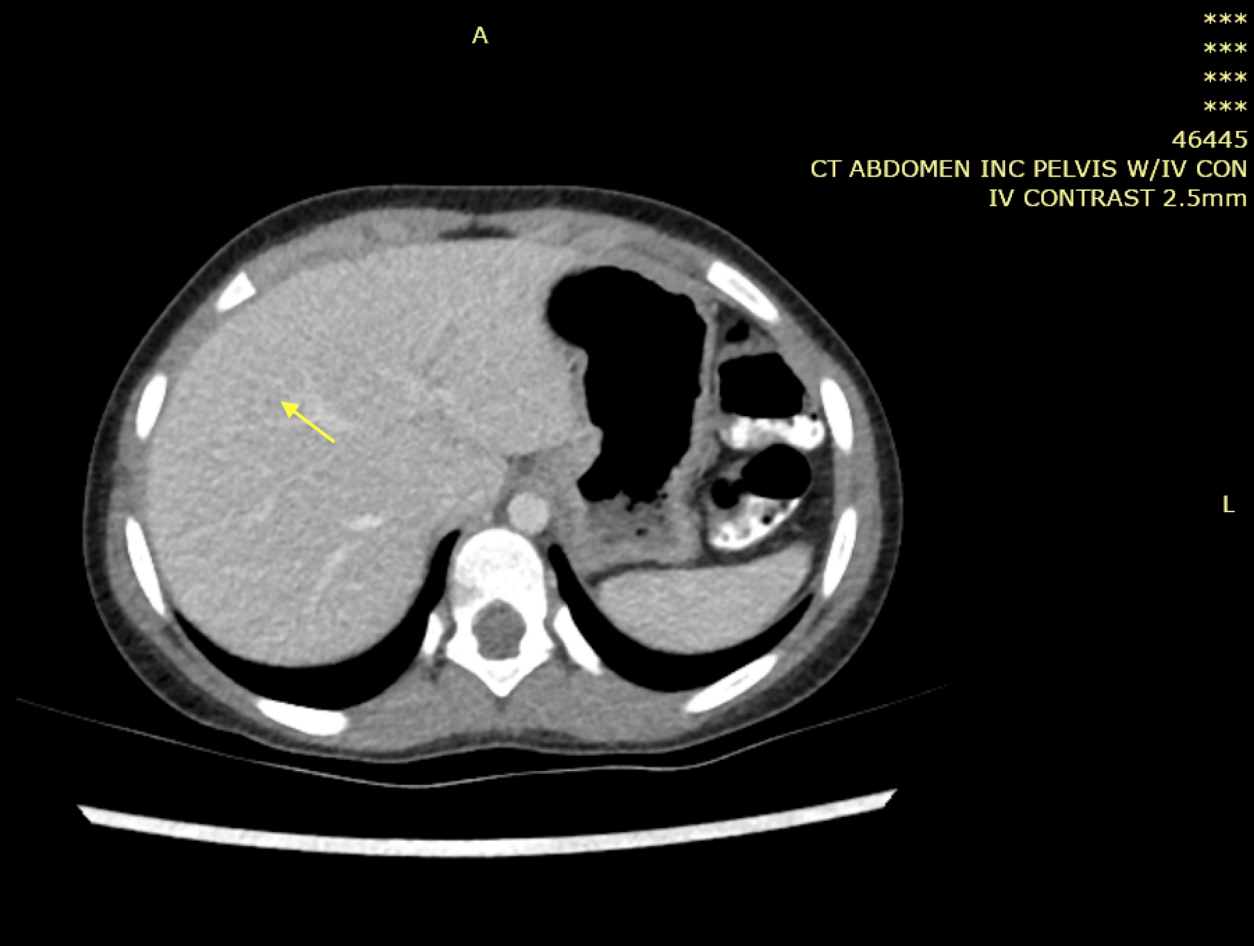
Computed tomography of the abdomen with IV contrast showing complete resolution of the two previously seen hepatic lesions (yellow arrow).

**Figure 10:**
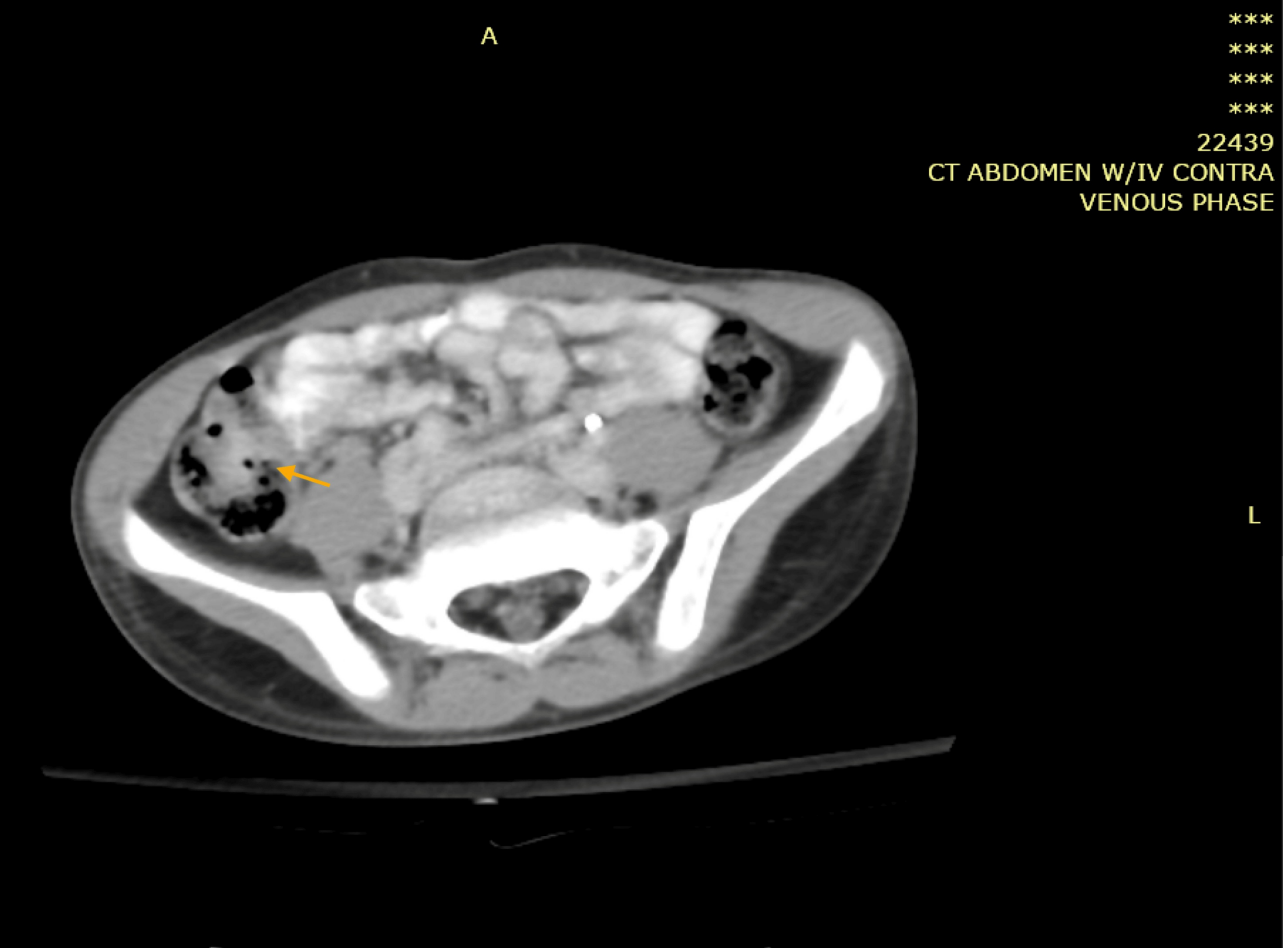
Computed tomography of the abdomen with IV contrast showing lymphadenopathy with almost complete resolution of the previously described wall thickening in the caecum and ascending colon region (orange arrow).

The patient initially was started on IV voriconazole for three weeks and then discharged on a full therapeutic course of oral itraconazole, which was continued for 11 months. He improved clinically, becoming asymptomatic, and all laboratory blood workup was normalized. He received regular follow up at the outpatient clinic. Follow-up CT abdomen with IV contrast showed complete resolution of the previous lesions (Figures 9, 10). After medication was stopped, the patient received regular follow-up in our clinic for clinical and radiological evaluation every six months, with no evidence of relapse.

Case 3

A 7-year-old female patient from Jizan presented to our hospital complaining of abdominal pain for one month, associated vomiting and constipation, decreased appetite, and fever lasting two weeks. Examination of the abdomen revealed localized tenderness, and positive rebound tenderness in the right iliac fossa, with unremarkable other systemic examinations. US abdomen showed a large soft-tissue mass lesion on the right iliac fossa measuring approximately 58 × 32 mm, suggesting enlarged suspected mesenteric lymph nodes associated with surrounding smaller lymph nodes, as well as para-aortic lymph nodes. The patient was admitted under pediatric surgery for further investigations. Initial lab workup showed microcytic hypochromic anemia, with normal leukocytes, eosinophilia, and thrombocytosis with elevated ESR and CRP (Table 3).

**Table 3:** Lab results showing microcytic hypochromic anemia, with normal leukocytes, eosinophilia, and thrombocytosis with elevated ESR and CRP. TWBC: total white blood cells, CRP: C-reactive protein, and ESR: erythrocyte sedimentation rate.

Lab test	Lab result	Reference range
Hemoglobin	11.9 g/dL	10-14 g/dl
TWBC	10.97 × 10^9 ^/L	6-17 × 10^9^/L
Eosinophil %	18.5%	-
Absolute eosinophilia	2.02 × 10^9 ^/L	0.2 - 1.9 × 10^9^/L
Platelets	580 × 10^9^/L	150 - 450 × 10^9^/L
CRP	24 mg/L	<5mg/L
ESR	51 mm/h	<10 mm/h

**Figure 11:**
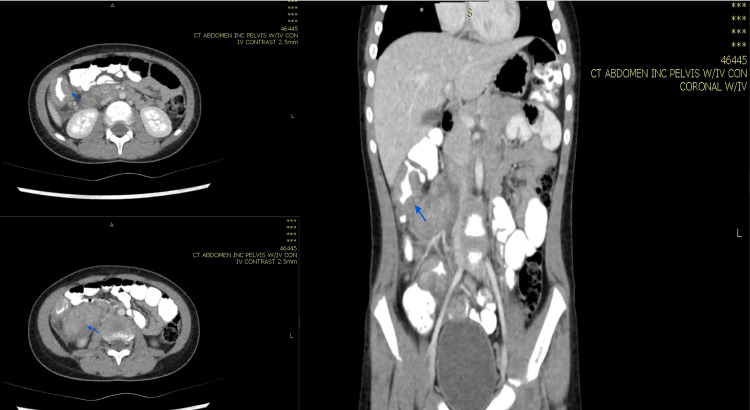
CT abdomen showing significant wall thickening of the ascending colon and terminal ileum with luminal narrowing (blue arrow), accompanied by multiple variable-sized lesions seen in the mesenteric fat of the right side of the abdomen suggestive of enlarged and pathological lymph nodes (the largest measuring approximately 40 × 23 mm).

CT abdomen was suggestive of either lymphoma or inflammatory bowel disease involving the large and small bowels (Figure 11).

**Figure 12:**
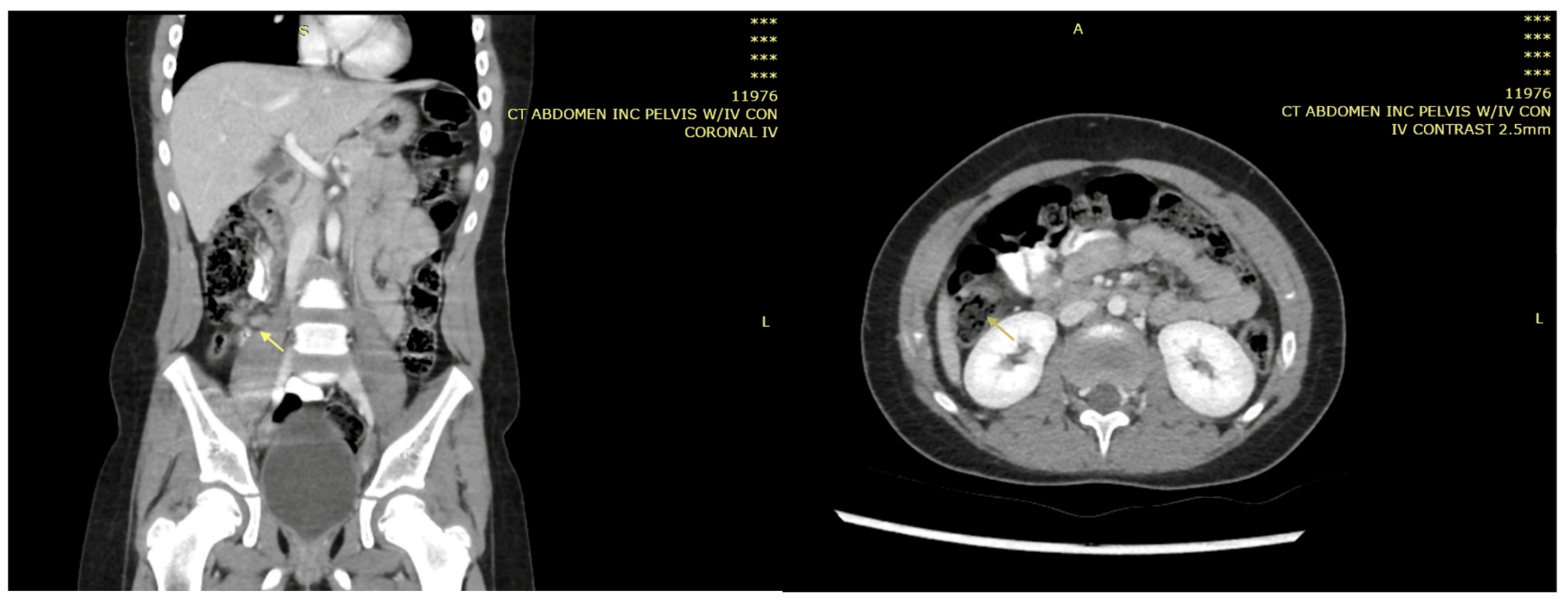
Repeat computed topography scan of the abdomen with IV contrast showing near total disappearance of the previously noted colonic and ileal masses (orange and yellow arrows), and now-mild residual thickening seen in the cecum and terminal ileum, with few residual lymph nodes.

US-guided true-cut biopsy of the retroperitoneal mass lesion was done. The histopathology results showed eosinophilic-rich lymphohistiocytic, plasmacytic infiltrate, occasional lymphoid follicles, epithelial cell granulomas, and some giant cells with PAS/PASD material suggestive of fungal hyphae inside, confirming the diagnosis of gastrointestinal basidiobolomycosis. The patient was started on IV voriconazole for two weeks. He improved clinically and became asymptomatic, and all laboratory parameters normalized. Therefore, he was shifted to a therapeutic dose of oral itraconazole for 10 months with regular follow-up in the infectious diseases clinic. A repeat CT scan of the abdomen with IV contrast showed near total disappearance of the previously noted colonic and ileal masses (Figure 12). In addition, repeat laboratory investigations performed 10 months after initiation of treatment were normal; therefore, the patient’s medication was stopped. He then received follow-up every three months in the infectious disease clinic.

## Discussion

Entomophthoromycosis is caused by *B. ranarum*, which represents a saprophytic fungus. Subcutaneous zygomycotic is the most common clinical form of basidiobolomycosis [3]. Also, it is a rare fungal infection caused by *B. ranarum, an* environmental saprophyte, that can cause significant disease and is occasionally fatal [7]. In 2018, Shreef et al. reported a large number of cases (18) in their multicentric study done in Saudi Arabia [8]. *B. ranarum* grows in warm and humid climates such as that of the southern region of Saudi Arabia, where most of the country’s cases develop. The mode of transmission remains unknown, where the portal of entry is believed to be the skin after loss of integrity by scratch or insect bite [3]. In addition, contaminated food from soil may be the main cause of infection, which may lead to involvement of the gut [8].

Herein, we presented three cases of basidiobolomycosis from the same area in Jizan, which is located in the southern region of Saudi Arabia. In such cases, the location of the patient may help to increase suspicion of basidiobolomycosis. However, the non-specific presentation, symptoms, and signs of gastrointestinal basidiobolomycosis may resemble those of conditions such as intestinal lymphoma, inflammatory bowel disease, abdominal tuberculosis, and sarcoidosis, resulting in misdiagnosis or delayed diagnosis, which may lead to morbidity.

For accurate diagnosis of basidiobolomycosis, physicians must be aware of endemic areas in Saudi Arabia, with a high index of suspicion in patients presenting with prolonged fever and long duration of abdominal pain with changes in bowel habits. Gastrointestinal basidiobolomycosis should also be part of differential diagnosis in patients with abdominal mass, weight loss, and other features of chronic infection or cancer, such as anemia thrombocytosis, leukocytosis, and high inflammatory markers (ESR and CRP).

Our three cases had similar presentations, including abdominal pain, prolonged fever, weight loss, abdominal mass, and changes in bowel habits, with no clear cause of infection. In addition, all three cases had laboratory findings of microcytic hypochromic anemia and leukocytosis with obvious eosinophilia, and high inflammatory markers. These results may help physicians to become more aware of basidiobolomycosis and diagnose it as early as possible to avoid complications that may necessitate surgical intervention. Doctors should also be aware that this rare condition, which is common in young children, is a disease of the skin or subcutaneous tissue that can be transmitted via loss of integrity of the skin [3].

Culture of the *B. ranarum* organism is the most accurate method for diagnosis, but diagnosis also can be made with typical findings of histopathological features of *B. ranarum*. These features include granulomatous inflammation rich in eosinophils, the Splendore-Hoeppli phenomenon, and broad-based hyphae on Gomori methenamine silver (GMS) [3]. The Splendor-Hoeppli phenomenon (also known as steroid bodies) was first described by Splendore in mucocutaneous tissue and internal organs, and by Hoeppli in cases of bilharziasis. Immune diffusion testing can also be used to reach a diagnosis of basidiobolomycosis by detecting immune response against the GMS [9]. In our case series, the diagnosis of gastrointestinal basidiobolomycosis was based on clinical presentation and radiological findings accompanied by unique and characteristic histopathological findings for *B. ranarum* in human tissue. All three patients were immunocompetent.

At present, appropriate treatment for gastrointestinal basidiobolomycosis has not been established. In some centers, a combination of surgical intervention for pathological removal and confirmation of diagnosis is used to prevent recurrence and minimize the duration of the antifungal course and its side effects. It was difficult to apply this approach in our cases, as they had multiple-site involvement (colon and liver abscess in case one; caecum, ascending colon with involvement of right and left hepatic lobes in case two; and ascending colon and terminal ileum in case three). In all cases, there were multiple lymph nodes involved; therefore, we preferred medical therapy initially, rather than initiating surgery, as the latter carries a high risk of complications [10]. To confirm their diagnosis, we used ultrasound-guided true-cut biopsy for histopathology, which is one method used to diagnose basidiobolomycosis.

All patients were started on IV voriconazole as a broad-spectrum antifungal agent (for two weeks in cases one and three, and three weeks in case two), as it is effective in reducing fungemia. All patients showed good response to treatment, including resolution of fever and abdominal pain, and improved appetite and general condition. The patients’ inflammatory markers improved, and their eosinophilia resolved. Thereafter, all three patients were changed to a therapeutic regimen of itraconazole and discharged in good condition with regular follow-up at the outpatient clinic for clinical and radiological monitoring.

In case 1, the patient was treated for one year, and repeated CT abdomen with IV contrast showed resolved lesions, and medication was stopped (Figures 4, 5). In case 2, the patient was treated for 11 months after repeated CT scan showed resolved lesions (Figures 9, 10). In case 3, the patient was treated for 10 months after repeated CT scan of the abdomen with IV contrast (Figure 12) showed near total disappearance of the previously noted colonic and ileal masses, as well as few residual lymph nodes. As mentioned previously, alerting physicians to the clinical presentation and endemic areas of this disease may lead to earlier diagnosis with better outcomes when treatment is started as early as possible.

## Conclusions

Gastrointestinal basidiobolomycosis can be easily misdiagnosed as malignancy, inflammatory bowel disease, or abdominal tuberculosis. As it is rare disease, its risk factors and clinical presentation are poorly understood. We reported this case series to improve pediatricians’ awareness of this disease, as they should consider it as a part of differential diagnosis in any patient with prolonged fever, abdominal pain, weight loss, eosinophilia, and high inflammatory marker, especially those coming from endemic areas. Early diagnosis and initiation of management can lead to excellent outcomes. We observe medical therapy even in cases of multiple lesions with abscesses, as our patients responded well to this approach. Histopathology has great value for diagnosis, as the fungal morphology and Splendore-Hoeppli phenomenon are characteristic histopathological features of the disease. CT abdomen can be used as an adjuvant for diagnosis and follow-up, as the latter depends on this imaging to determine when to stop medication. This case series can be used as a reference for gastrointestinal basidiobolomycosis management guidelines.
